# The complete mitochondrial genome of *Ischiodon scutellaris* (Diptera: Syrphidae: Syrphinae)

**DOI:** 10.1080/23802359.2025.2579080

**Published:** 2025-10-31

**Authors:** Jingqiang Zhou, Yi Li, Xuefei Cai, Feng Li, Hong-Peng Xiao, Guo-Hua Huang

**Affiliations:** ^a^ College of Plant Protection, Hunan Provincial Key Laboratory for Biology and Control of Plant Diseases and Insect Pests, Hunan Agricultural University, Changsha, China; ^b^ Amway (China) Botanical R&D Center, Wuxi, China; ^c^ Amway (Shanghai) Innovation & Science Co., Ltd, Shanghai, China

**Keywords:** *Ischiodon scutellaris*, mitogenome, phylogenetic analysis

## Abstract

This study reports the first complete mitochondrial genome of *Ischiodon scutellaris*. The mitochondrial genome is 15,815 bp in length, with an A + T content of 80.7%. The genome contains 13 protein-coding genes (PCGs), 22 tRNAs, 2 rRNAs, and a control region. All PCGs initiate with ATN (G/C/T), except *COX1*and *ND1*(TTG). Three PCGs (*COX1*, *ATP6*, *ND5*) terminate with an incomplete T stop codon. Phylogenetic analysis based on 13 PCG sequences using maximum likelihood revealed that *I. scutellaris* forms a sister clade with *Scaeva affinis* and clusters closely with multiple Eupeodes species. This study advances understanding of syrphid mitochondrial genomics and evolutionary relationships.

## Introduction

Syrphidae, a major insect family within the order Diptera, encompasses approximately 6000 species classified into 200 genera (Mengual et al. 2008, 2015), with 465 species from 80 genera recorded in China alone (Huo et al. 2002)*. Ischiodon scutellaris* (Fabricius, 1805), a species belonging to the family Syrphidae, represents a significant biological control agent due to its predatory nature against aphids. This study provides the genomic foundation for future exploration of its biological control potential. The larval stage demonstrates remarkable predation capacity, consuming up to 278 aphids per individual, and in the Fuzhou region, this species completes 6–7 generations annually, overwintering and estivating as pupae in soil (She et al. 1994). Our study presents the first complete mitochondrial genome assembly of *I. scutellaris*. Phylogenetic reconstruction using maximum likelihood analysis based on concatenated sequences from 13 protein-coding genes elucidates the evolutionary relationships within this taxon.

## Materials and methods

**Figure 1.**
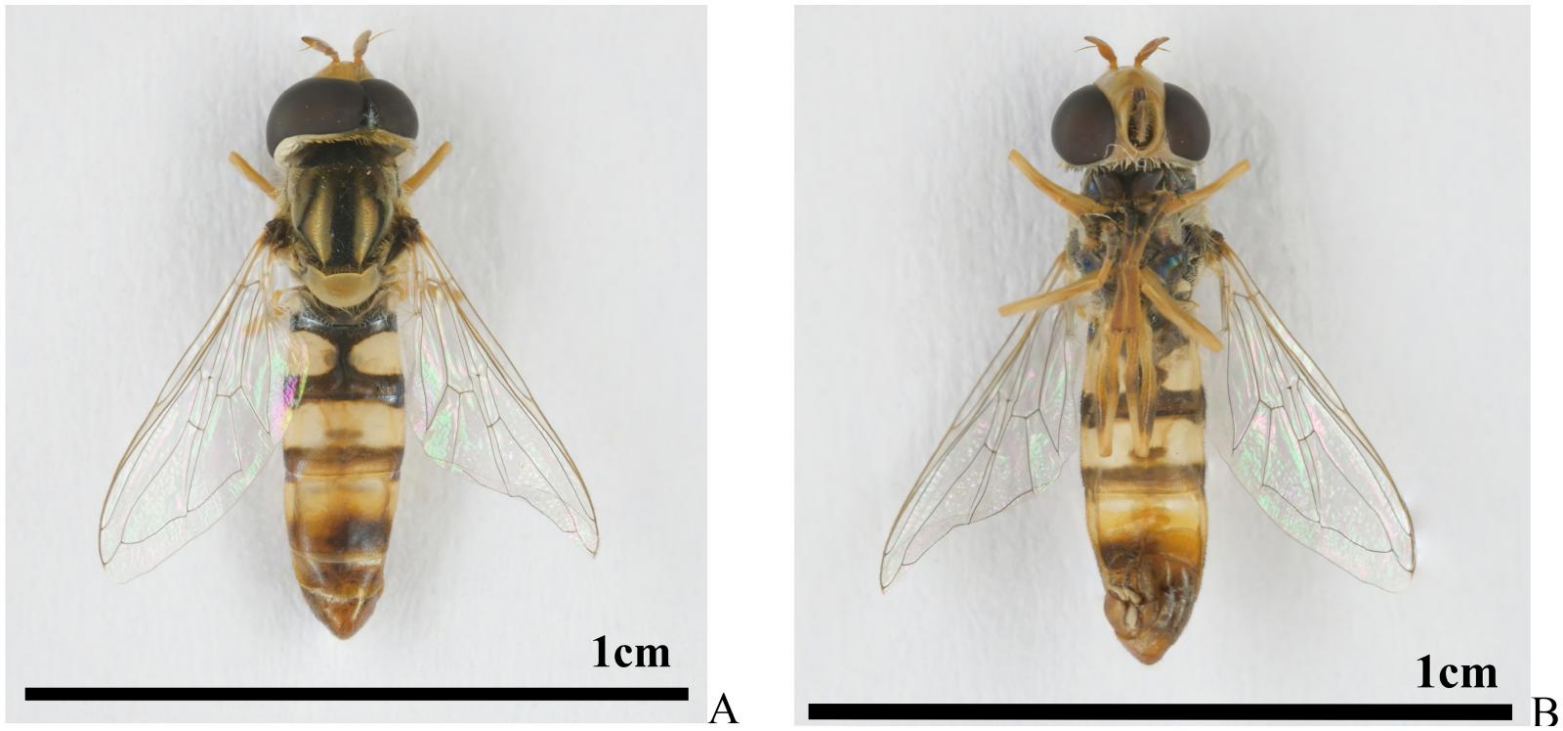
The adult of *I. scutellaris*. (A) The dorsal view; (B) the ventral view. Photoed by Jing-Qiang Zhou.

Specimens of *I. scutellaris* used in this study were collected on 10 September 2024 by Zhou JingQiang at the Broccoli Selenium-enriched Ecological Planting Base, Longgui Township, Shaoguan City, China (24°44′N, 113°25′E). Adult specimens measure 8–10 mm in body length with glabrous compound eyes and a metallically iridescent blue-gray mesoscutum. The integument bears yellowish setae, while the abdominal tergites display diagnostic coloration: tergite II exhibits paired, distally separated yellow maculae; tergites III and IV each bear an anterior transverse yellow band; tergites IV and V feature yellow posterior margins against a predominantly brown to yellowish-brown ground color. The specimens (Figure 1) and genomic DNA are kept at the Insect Collections of Hunan Agricultural University, Changsha, China (voucher code: HAUHL141957; contact person: Guo-Hua Huang, ghhuang@hunau.edu.cn).

**Figure 2.**
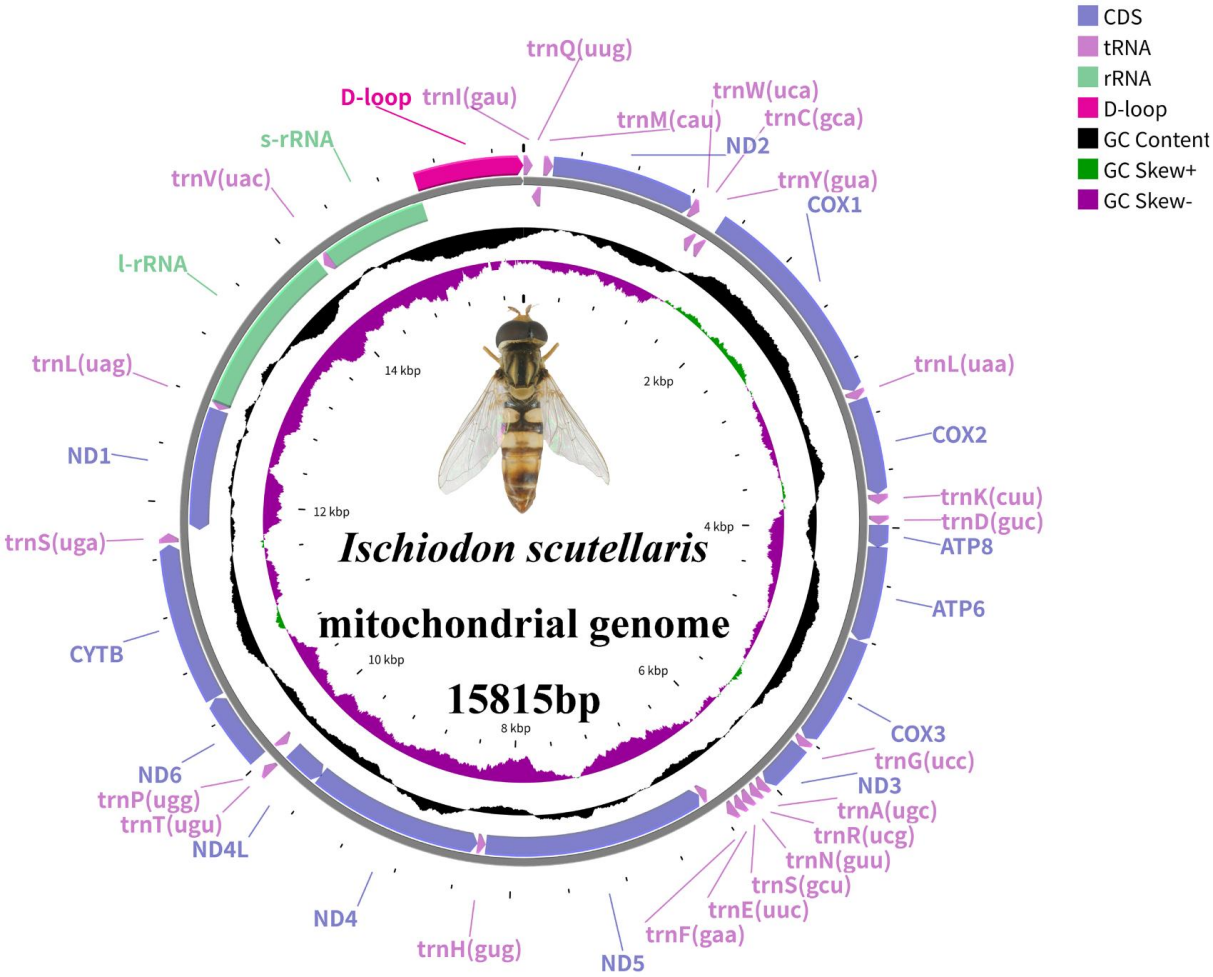
Mitogenome pattern map of *I. scutellaris*. Genes inside the gray circle are coded in the minority strand (N-strand); genes outside the black circle are coded in the majority strand (J-strand).

The DNA was extracted from the thorax of *I. scutellaris* adults using the TIANamp Genomic DNA Kit (DP304; TIANGEN). Genomic DNA was enzymatically fragmented to 350 bp, end-repaired, A-tailed, and ligated with Illumina adapters. Libraries were quantified and size-selected before paired-end sequencing (2 × 150 bp) on the Illumina NovaSeq X Plus platform (Berry Genomics, Beijing) using NovaSeq6000 S4 Reagents. Raw reads were filtered using Fastp (Chen et al. 2018). The complete mitochondrial genome of *I. scutellaris* was assembled using NOVOPlasty (Dierckxsens et al. 2017) and GetOrganelle (v1.7.6) (Jin et al. 2020). The average sequencing coverage depth for the mitochondrial genome was 4582x, with a maximum of 15,389x and a minimum of 11x. Most positions showed high coverage (usually >3000x), indicating that the sequencing data were robust and reliable. The read coverage depth map for *I. scutellaris* is presented in Supplementary Figure S1. The mitochondrial genome was annotated using MITOS (Bernt et al. 2013), and adjustments to the mitochondrial genome of *I. scutellaris* were made using Geneious (Kearse et al. 2012). Finally, CGview was used for visualization (Grant et al. 2023), drawing a circular map of the mitochondrial genome (Figure 2) (https://proksee.ca/).

Nineteen published Syrphinae species, along with two Eristalinae species serving as outgroups, were retrieved from the NCBI database (Table S1). Thirteen protein-coding genes (PCGs) sequences were manually extracted and concatenated using Geneious Prime (v2024.0.5). Multiple sequence alignment was performed using MAFFT (v1.5.0) (Katoh and Standley 2013), the most suitable model was calculated by ModelFinder (Kalyaanamoorthy et al. 2017). Finally, IQ-TREE (v2.4.0) (Minh et al. 2020) was used to construct the maximum likelihood (ML) phylogenetic tree, with a bootstrap value of 1000. The molecular evolution model was GTR+ F + I + R2. The phylogenetic tree was visually refined using FigTree (v1.4.4) and iTOL (https://itol.embl.de/) (Ivica and Peer 2024).

## Results

### Mitogenomic characteristics

The complete mitochondrial genome sequence of *I. scutellaris* was sequenced (GenBank accession: PV021573). with base composition [A] = 41.1%, [G] = 8.3%, [C] = 11.0%, and [T] = 39.6%. The genome comprises 37 genes: 13 protein-coding genes (PCGs), 22 tRNAs, 2 rRNAs, and one control region. Comprehensive annotation is documented in Supplementary Table S2. Among the 13 PCGs, 11 initiate with ATN (N: G, C, or T), both *COX1* and *ND1* initiate with TTG. Ten PCGs terminate with TAA, while *COX1*, *ATP6*, and *ND5* end with a single T residue (incomplete stop codon). Twenty-three genes are located on the heavy chain and 14 on the light chain. The 22 tRNA genes are 64–72 bp in length, 12S rRNA and 16S rRNA are 822 bp and 1340 bp, respectively, and the control region is 776 bp in length. Intergenic spacers range from 1 bp to 80 bp, occurring between 19 gene pairs. Eight gene pairs exhibit overlapping regions (1–19 bp), totaling 43 bp of sequence overlap.

### Phylogenetic analysis

**Figure 3.**
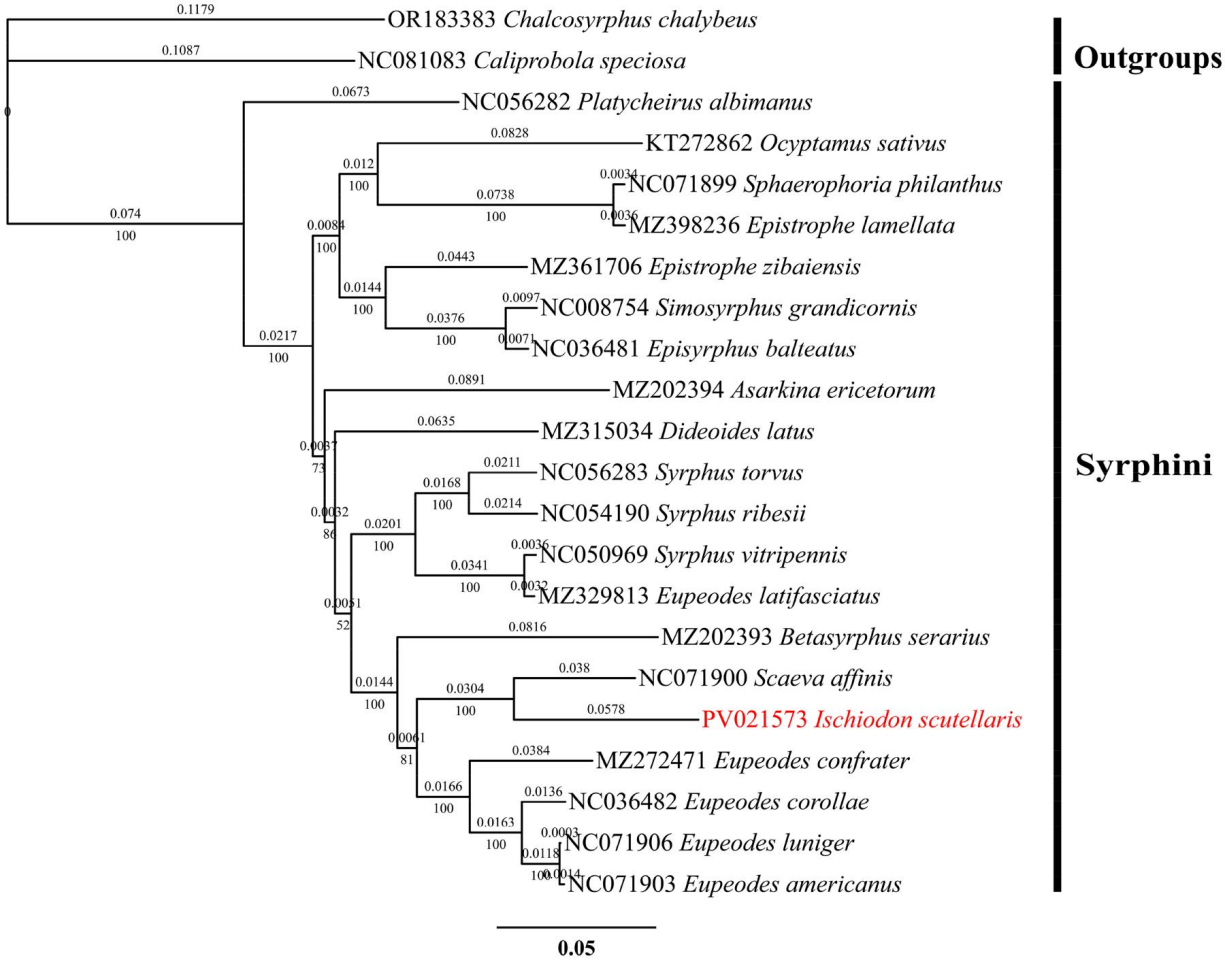
Maximum likelihood (ML) tree of 22 species within the tribe Syrphinae based on 13 PCGs of the mitogenome with two Eristalinae species as outgroups. The numbers at the nodes represent the bootstrap values and genetic distances. The following sequences were used: OR183383 *Chalcosyrphus chalybeus* (Wong et al. 2023), NC081083 *Caliprobola speciosa* (Wong et al. 2023), NC056282 *Platycheirus albimanus* (Direct submission), KT272862 *Ocyptamus sativus* (Junqueira et al. 2016), NC071899 *Sphaerophoria philanthus* (Direct submission), MZ398236 *Epistrophe lamellate* (Direct submission), MZ361706 *Epistrophe zibaiensis* (Direct submission), NC008754 *Simosyrphus grandicornis* (Cameron et al. 2007), NC036481 *Episyrphus balteatus* (Pu et al. 2017a), MZ202394 *Asarkina ericetorum* (Direct submission), MZ315034 *Dideoides latus* (Direct submission), NC056283 *Syrphus torvus* (Direct submission), NC054190 *Syrphus ribesii* (Chen et al. 2021), NC050969 *Syrphus vitripennis* (Liu et al. 2020), MZ329813 *Eupeodes latifasciatus* (Direct submission), MZ202393 *Betasyrphus serarius* (Direct submission), NC071900 *S. affinis* (Direct submission), PV021573 *Ischiodon scutellaris* (this study), MZ272471 *Eeupeodes confrater* (Direct submission), NC036482 *Eupeodes corollae* (Pu et al. 2017b), NC071906 *Eupeodes luniger* (Direct submission), NC071903 *Eupeodes americanus* (Direct submission).

We selected mitochondrial genome sequences of 22 species, including species from the subfamilies Eristalinae and Syrphinae, with two species of Eristalinae as outgroups. A maximum likelihood evolutionary tree (ML) was constructed for phylogenetic analysis using 13 PCGs from these mitochondrial genomes (Figure 3). The tree shows that *I. scutellaris* and *S. affinis* formed a branch with a distance of 0.0304 between them. The bootstrap support value for this branch is 100%, indicating that *I. scutellaris* and *S. affinis* are supported as sister groups. Additionally, *I. scutellaris* is closely related to several species of Eupeodes, with 100% bootstrap support for this relationship, and together they constitute a larger clade.

## Discussion and conclusion

This study presents the first complete mitochondrial genome (mitogenome) sequencing of the syrphid predator *I. scutellaris* (Diptera: Syrphidae), with a total length of 15,815 bp. The mitogenome comprises 37 genes, including 13 protein-coding genes (PCGs), 22 tRNA genes, 2 rRNA genes, and a control region, exhibiting a typical dipteran A + T bias (80.7%) (Zhang et al. 2013). Mitogenomic sequences provided well-supported phylogenetic resolution for Syrphidae systematics across subfamily and tribe divergences (Wong et al. 2023). Phylogenetic reconstruction using maximum likelihood (ML) analysis based on concatenated sequences of 13 PCGs revealed that *I. scutellaris* forms a strongly supported sister clade (100% bootstrap support) with *S. affinis*. It also clusters with multiple Eupeodes species in a monophyletic group (100% bootstrap support), thereby confirming its taxonomic placement within Syrphidae and providing insights into its ecological adaptation mechanisms. Furthermore, our ML analysis strongly supports a sister-group relationship between the (*I. scutellaris* + *S. affinis*) clade and the *Eupeodes corollae* clade; this result aligns with prior phylogenetic research (Wu et al. 2024; Zhao et al. 2024). These findings elucidate the mitogenomic architecture and evolutionary relationships of *I. scutellaris*, clarifying its phylogenetic position relative to congeners. The taxonomic framework established in this study may provide a theoretical basis for future natural enemy insect management strategies; however, practical applications require further validation through field trials.

## Supplementary Material

Table S1.pdf

Table S2.pdf

Figure S1.jpg

## Data Availability

The genome sequence data that support the findings of this study are openly available in GenBank of NCBI under the accession number PV021573. The associated BioProject, SRA, and BioSample numbers are PRJNA1227853, SRR32520600, and SAMN46992965, respectively.
