# Supplementary material for: The complete mitochondrial genome of Ischiodon scutellaris (Diptera: Syrphidae: Syrphinae)
Source: Mitochondrial DNA B Resour. 2025 Oct 31;10(12):1078–82. doi: 10.1080/23802359.2025.2579080 (PMC12581767; doi:10.1080/23802359.2025.2579080)
Supplement: Table S1.pdf [file TMDN_A_2579080_SM3776.pdf]

The Diptera mitochondrial genomes used for phylogenetic inference		
**Accessionnumber**	**Species**	**Reference**
OR183383	*Chalcosyrphus chalybeus*	Wong et al. 2023a
NC081083	*Caliprobola speciosa*	Wong et al. 2023b
NC056282	*Platycheirus albimanus*	Direct submission
KT272862	*Ocyptamus sativus*	Junqueira et al. 2016
NC071899	*Sphaerophoria philanthus*	Direct submission
MZ398236	*Epistrophe lamellate*	Direct submission
MZ361706	*Epistrophe zibaiensis*	Direct submission
NC008754	Simosyrphus grandicornis	Cameron et al. 2007
NC036481	*Episyrphus balteatus*	Pu et al. 2017a
MZ202394	*Asarkina ericetorum*	Direct submission
MZ315034	*Dideoides latus*	Direct submission
NC056283	*Syrphus torvus*	Direct submission
NC054190	*Syrphus ribesii*	Chen et al. 2021
NC050969	*Syrphus vitripennis*	Liu et al. 2020
MZ329813	*Eupeodes latifasciatus*	Direct submission
MZ202393	*Betasyrphus serarius*	Direct submission
NC071900	*Scaeva affinis*	Direct submission
PV021573	*Ischiodon scutellaris*	This study
MZ272471	*Eupeodes confrater*	Direct submission
NC036482	*Eupeodes corollae*	Pu et al. 2017b
NC071906	*Eupeodes luniger*	Direct submission
NC071903	*Eupeodes americanus*	Direct submission
