# Supplementary material for: The complete mitochondrial genome of Ischiodon scutellaris (Diptera: Syrphidae: Syrphinae)
Source: Mitochondrial DNA B Resour. 2025 Oct 31;10(12):1078–82. doi: 10.1080/23802359.2025.2579080 (PMC12581767; doi:10.1080/23802359.2025.2579080)
Supplement: Table S2.pdf [file TMDN_A_2579080_SM3775.pdf]

Annotation table of Ischiodon scutellaris mitogenome

Gene	Position(bp)	Length(bp)	Direction	Intergenic nucleotides	start/stop codons	AT%
trnI	1-66	66	J	-3		80.3%
trnQ	64-132	69	N	11		78.3%
trnM	144-212	69	J	0		72.5%
ND2	213-1238	1026	J	-2	ATT/TAA	85.6%
trnW	1237-1304	68	J	-1		83.8%
trnC	1304-1369	66	N	22		81.8%
trnY	1392-1457	66	N	18		77.3%
COX1	1476-3009	1534	J	0	TTG/T	72.1%
trnL	3010-3075	66	J	2		75.8%
COX2	3078-3761	684	J	0	ATG/TAA	75.7%
trnK	3762-3832	71	J	80		70.4%
trnD	3913-3980	68	J	0		91.2%
ATP8	3981-4142	162	J	-7	ATT/TAA	87.0%
ATP6	4136-4811	676	J	2	ATG/T	75.9%
COX3	4814-5602	789	J	3	ATG/TAA	73.6%
trnG	5606-5672	67	J	0		85.1%
ND3	5673-6026	354	J	3	ATT/TAA	81.9%
trnA	6030-6097	68	J	0		82.4%
trnR	6098-6161	64	J	7		75.0%
trnN	6169-6235	67	J	0		80.6%
trnS	6236-6302	67	J	3		79.1%
trnE	6306-6372	67	J	27		91.0%
trnF	6400-6466	67	N	3		79.1%
ND5	6470-8201	1732	N	0	ATT/T	81.1%
trnH	8202-8267	66	N	-1		86.4%
ND4	8267-9607	1341	N	-7	ATG/TAA	80.8%
ND4L	9601-9897	297	N	2	ATG/TAA	83.2%
trnT	9900-9964	65	J	0		83.1%
trnP	9965-10030	66	N	2		81.8%
ND6	10033-10557	524	J	3	ATC/TAA	85.3%
CYTB	10561-11697	1137	J	13	ATG/TAA	75.8%
trnS	11711-11778	68	J	16		82.4%
ND1	11795-12742	948	N	1	TTG/TAA	80.2%
trnL	12744-12808	65	N	-19		84.6%
16S rRNA	12790-14129	1340	N	19		84.3%
trnV	14149-14220	72	N	-3		79.2%
12S rRNA	14218-15039	822	N	0		83.5%
control region D-loop	15040-15815	776	J			92.7%
