# Supplementary figures and images for: The complete mitochondrial genome of Ischiodon scutellaris (Diptera: Syrphidae: Syrphinae)
Source: Mitochondrial DNA B Resour. 2025 Oct 31;10(12):1078–82. doi: 10.1080/23802359.2025.2579080 (PMC12581767; doi:10.1080/23802359.2025.2579080)

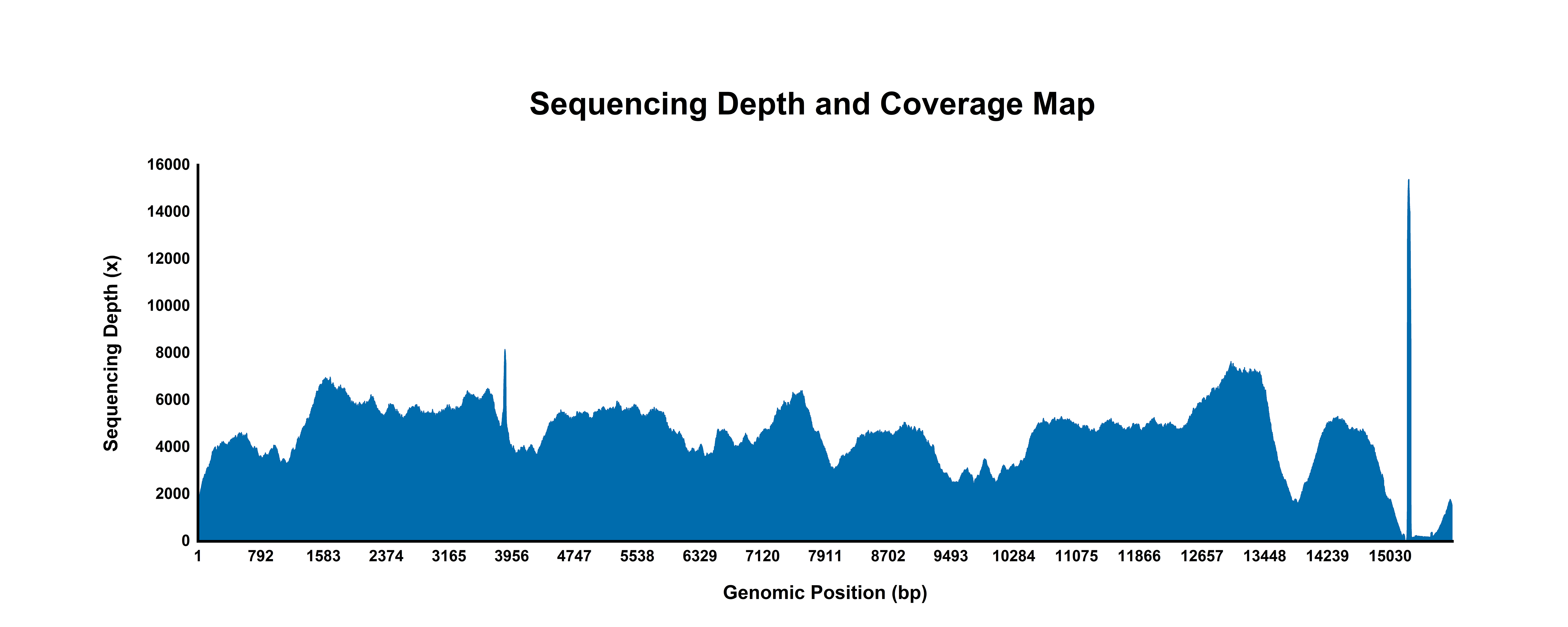

Supplement: Figure S1.jpg [file TMDN_A_2579080_SM3774.jpg]
